# To the Medical Profession of Illinois

**Published:** 1860-07

**Authors:** S. T. Trowbridge

**Affiliations:** Chairman; Decatur, Ill.


### TO THE MEDICAL PROFESSION OF ILLINOIS.

The Committee on Practical Medicine, appointed at the last sitting of the Illinois State Medical Society, would respectfully invite the co-operation of its members, and the profession of the the State in general, in furnishing material for their report, hoping each one will feel himself individually called upon to forward to the Chairman of this Committee, such observations, of a practical nature, pertaining to disease, of either epidemic, sporadic, or other nature, upon which he may feel inclined to treat. We ask of each to give his own views, in his own way, upon whatever subject would legitimately take a place in this report, and that the same may be submitted to us at as early a day as the 1st of February, 1861.

S. T. TROWBRIDGE, Chairman.

Decatur, Ill.

